# Septic thrombophlebitis of the superior mesenteric vein and multiple liver abscesses in a patient with Crohn's disease at onset

**DOI:** 10.1186/1471-230X-7-22

**Published:** 2007-06-12

**Authors:** Mariam Aguas, Guillermo Bastida, Pilar Nos, Belen Beltrán, Jose Luis Grueso, Julio Grueso

**Affiliations:** 1 Gastroenterology Department, Hospital La Fe, Avenida Campanar, 21. 46009 Valencia, Spain

## Abstract

**Background:**

Portal-mesenteric vein thrombosis, pylephlebitis and liver abscesses are rare complications of inflammatory bowel disease (IBD). The purpose of this case report is to relate an unusual presentation of CD in order to show how conservative treatment could be an appropriate option as a bridge to the surgery, in patients with septic thrombophlebitis and multiple liver abscesses with CD.

**Case presentation:**

We report a case of a 25-year-old man with Crohn's disease (CD) who developed a superior mesenteric venous thrombosis, multiple liver abscesses and pylephlebitis, diagnosed through abdominal ultrasound and an abdominal computed tomography (CT) scan. The patient was successfully treated with conservative treatment consisting of intravenous antibiotics, subcutaneous anticoagulation and percutaneous catheter drainage of liver abscesses.

**Conclusion:**

We reported an unnusual case of pylephlebitis in CD. Until now this association has not been reported in adult patients at onset. We hypothesise that the infection developed as a result of mucosal disease and predisposed by corticoid therapy. Adequated management was discussed.

## Case presentation

A 25-year-old white male smoker patient was admitted to our hospital due to fever and right lower quadrant abdominal pain. Six years previously he suffered from perianal fistulas and underwent perianal surgery including drainage and fistulectomy. Two years later CD of the terminal ileum was diagnosed with an upper gastrointestinal barium contrast study and an endoscopic examination (colonoscopy with multiple biopsies). The patient was treated with mesalazine and was in clinical remission most of the time, except for occasional episodes of mild watery diarrhea without fever.

Two months prior to admission the patient had visited the outpatient clinic with abdominal pain and diarrhoea. A physical examination revealed a palpable mass in the right lower quadrant and laboratory analysis showed elevation of acute phase reactants. A gastrointestinal barium contrast was performed and confirmed the presence of a stenosis in the terminal ileum. The patient was treated with oral prednisone (0.5 mg/kg followed by a standard weaning strategy), to which he responded well.

Two months later the patient was attended to in the emergency department with a seven day history of abdominal pain, fever and malaise. At the time he was still taking 5 mg of prednisone by month per day. A physical examination showed a heart rate of 120/min, blood pressure of 130/65 mmHg and temperature of 39°C. His skin, chest and heart were normal. The abdomen was soft, non-distended, slightly tender in the right lower quadrant and with hypoactive bowel sounds. A rectal examination gave a normal result. Laboratory results on admission are listed in table 1. Thrombophilia studies were normal and included fasting plasma levels of protein C, protein S, anti-thrombin III, lupus anticoagulant, anti-cardiolipin and anti-phospholipid antibodies, antinuclear antibodies, and genetic testing for the factor V Leiden. Chest and abdominal X-rays were normal. An ultrasound abdominal examination revealed multiple hypoechoic nodular lesions in both lobes of the liver suggestive of abscesses.

**Table 1:** Laboratory findings on patient admission.

Leukocyte count × 10^3^/μL	15.7 [4.8–10.8]
Neutrophil count × 10^3^/μL	13.7 [1.4–5.6]
Hemoglobin in g/dL	10.9 [14–18]
Platelet count × 10^3^/μL	457 [125–400]
Erythrocyte sedimentation rate mm/h	100 [<15]
C-reactive protein in mg/dL	479 [0–8]
Fibrinogen in mg/dl	661 [170–400]
Albumin in g/L	2.2 [3.5–5]
Alkaline phosphatase in IU/L	329 [40–130]
Gamma-glutamyl transferase in IU/L	524 [11–50]
Total bilirrubin in mg/dL	2.2 [0.1–1.1]
Direct bilirrubin in mg/dL	1.1 [0.0–0.3]
Lactate deshydrogenase in mlU/ml	710 [0–480]
Prothrombin time in seconds	15.8" [11.5–13.7]
Coagulation parameters	
Protein S, protein C, anti-thrombin III Anticardiolipin antibodies	Normal Negatives

**Figure 1:**
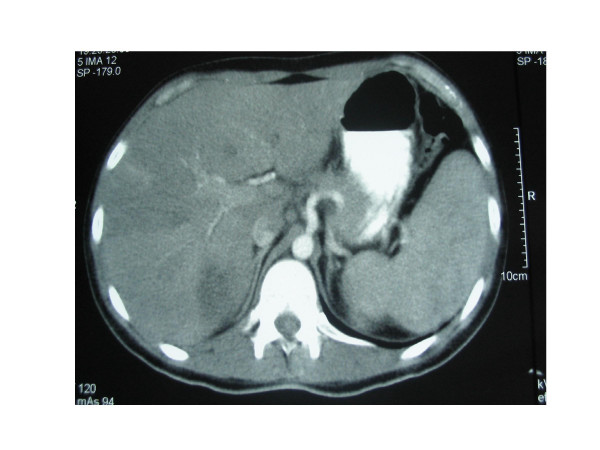
Abdominopelvic contrast-enhanced computed tomography. Multiple liver abscesses and mesenteric partial thrombosis.

**Figure 2:**
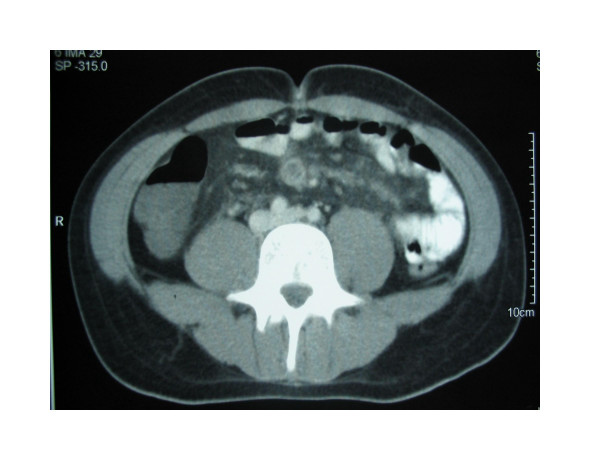
Abdominopelvic contrast-enhanced computed tomography. Multiple liver abscesses and mesenteric partial thrombosis.

Abdominopelvic contrast-enhanced computed tomography confirmed the existence of multiple liver abscesses and mesenteric partial thrombosis (Figure 1, 2). The latter finding was confirmed by abdominal ultrasound with Doppler. Computed tomography also revealed a dilated small bowel with several thick-walled loops of terminal ileum and ruled out an abscess of the terminal ileum.

The patient was heparinized (sc enoxaparin 70 mg q12 h) and treated with iv pipericillin/tazobactam 4 g q6 h and metronidazole 500 mg q6 hr for 3 weeks. He received total enteral nutrition and the corticosteroids were withdrawn within the first days of treatment.

The patient underwent an ultrasound percutaneous-guided needle aspiration and percutaneous drainage of the largest abscess. Culture of the fluid revealed *Peptostreptococcus spp *and *Propinibacterium acnes*, which were both sensitive to the antibiotics administered. All blood cultures tested negative.

**Figure 3:**
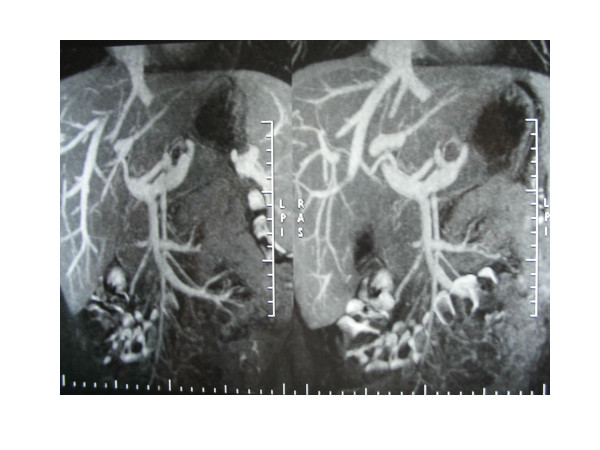
Magnetic resonance imaging. Decrease in the size and number of liver abscesses and no signs of thrombosis.

Following admission, the patient's clinical evolution was encouraging, although he was febrile until the abscesses were drained. Follow-up angio magnetic resonance imaging (MRI) on hospitalization day 20th demonstrated a marked decrease in the size and number of liver abscesses and no signs of thrombosis (Figure 3). After 1 month, the patient was discharged and prescribed po amoxicillin-clavulanate 2 g q12 h and metronidazole 250 mg q8 h for 4 weeks and sc enoxaparin 40 m q12 until he was operated on 5 months later. Prior to surgical intervention, a CT scan showed complete resolution of the liver abscesses and recanalization of the superior mesenteric vein. The patient underwent a surgical resection of the terminal ileum and cecum. Pathological report confirmed the presence of structure with no fistula. After two years follow-up the patient remains in goog health.

## Conclusion

Until now the association of pylephebitis and liver abscesses with CD has only been reported in a paediatric patient [1]. A reported case of a young man with pylephlebitis and liver abscesses associated said with CD confirmed was treated successfully with conservative therapy followed by surgery.

Liver abscesses are a rare complication of IBD, and most cases described to date have been in patients with CD [2-4]. Although their etiology is not well known, several factors have been implicated as representing a predisposition to developing liver abscesses. Abdominal surgery, long-term treatment with steroids or metronidazole, inflammatory and perforating diseases, fistulae and intra-abdominal abscesses and malnutrition are among those factors, but there is a lack of evidence to support their association with liver abscesses [2,4]. In the literature most patients with liver abscesses have a long-standing history of IBD although reports of CD presenting de novo with hepatic abscess have been presented [4-6]. The only predisposing factor in the patient whose case study we present here was the steroid treatment he was undergoing at the time of diagnosis.

Clinical manifestations of liver abscesses are variable, but certain symptoms are more common, such as fever, anorexia, weight loss and abdominal pain. However, their diagnosis is often incorrect, as their clinical presentation can resemble an exacerbation of CD.

Standard treatment of liver abscesses consists of broad spectrum antibiotics and percutaneous drainage. Although the hepatic abscesses were resolved with conservative treatment, a subsequent surgical resection of the affected ileum is mandatory.

Thromboembolic complications of IBD are well-recognized [7]. These complications can be caused by coagulation abnormalities induced by inflammation or steroid therapy [8]. Smoking has also been shown to cause morphological injury in endothelial cells [9]. Other risk factors to have been associated with a thrombotic tendency are active bowel disease, a history of clotting complications, prior abdominal surgery and sepsis, particularly in the perioperative phase. In the present case we ruled out an underlying hypercoagulable condition, and so it is likely the mesenteric thrombosis was related to the CD (bowel inflammation and steroid use).

The combination of infection and portal-mesenteric thrombosis can lead to the development of pylephlebitis. Portal pylephlebitis – defined as septic thrombophebitis of the portal vein or one of its tributaries – is an uncommon complication of suppurative infections, either in the region drained by the portal system or in strictures contiguous to the portal vein. Diverticulitis and appendicitis are the most common causes of pylephlebitis, but it is also attributed to IBD [10]. CD was first reported as a cause of pylephlebitis in 1946. Since then, a total of six further cases of pylephlebitis in patients with CD have been reported, and in all but one of these cases CD was diagnosed prior to the development of pylephlebitis [10-12]. The clinical features of these patients have generally been non-specific. A diagnosis of pylephlebitis should be considered in any patient with evidence of intraabdominal infection and high grade bacteremia. Abdominal ultrasound with colour Doppler is crucial in establishing the diagnosis of this condition. However, CT scan is more sensitive than ultrasound for detecting a thrombus within the splenic and mesenteric veins, and therefore should be the preferred imaging technique for detecting both thrombi and pericolonic abscesses [10]. It should be pointed out that the detection of thrombus in the portal vein or one of its tributaries does not necessarily indicate septic thrombophebitis. Treatment in these circumstances is based on a strategy of antibiotics and eradication of the septic focus. A broad spectrum antibiotic coverage is crucial, but the appropriate timing of their administration is less clear. Given the frequency of hepatic abscesses as a complication of pylephlebitis, and since they may not be visualized in a CT scan (with or without drainage), a minimum of 6 weeks antibiotic therapy seems prudent [13,14]. The role of anticoagulation or thrombolytic therapy in the treatment of pylephlebitis in the setting of IBD has not been standardized, but systemic anticoagulation for a minimum of 6 months has been recommended. Delay in initiating the appropriate treatment can result in unfavourable outcomes such as liver abscess formation (as occurred in our case), complications due to portal hypertension or venous infarction of the bowel [15].

## Competing interests

The author(s) declare that they have no competing interests.

## Authors' contributions

MS and GR have participated in the acquisition of data, have written the manuscript and have given final approval of the version to be published. Other authors have been involved in revising the manuscript critically for important intellectual content and have given final approval of the version to be published.

All authors have read and approved the final manuscript.

## Pre-publication history

The pre-publication history for this paper can be accessed here:


